# Glycovariants of CA-125 in the early detection of ovarian cancer

**DOI:** 10.64898/2026.09.11.26362728

**Published:** 2026-09-14

**Authors:** Andrew J. Vickers, Olle Melander, Kara Long Roche, Katri Kuningas, Marjut Helle, Kaisa Huhtinen, Leena Kokko, Anders Dahlin, Hans Lilja

## Abstract

**Importance:**

Two randomized trials have shown that screening with CA-125 is not of value for reducing ovarian cancer mortality. This appears to be because changes in CA-125 occur too late in the disease process to allow for surgical cure of early-stage disease.

**Objective:**

To determine whether glycovariants of CA-125 could detect ovarian cancer years before clinical diagnosis.

**Design:**

Case-control study of cryopreserved blood samples and data from the population-based Malmö Diet and Cancer cohort.

**Setting:**

Population-based study in Sweden.

**Participants:**

Serum was available for 17,297 women aged 44 – 73 collected at baseline in 1991-1996 and from 2,168 women re-sampled in 2007-2012. Participants’ records were linked to the Swedish cancer registry to identify 82 women with incident ovarian cancer diagnosed up to 10 years after blood draw. Cases were matched 1:3 with controls by age and venipuncture date.

**Exposure:**

Serum was measured for CA-125 glycovariants Sialyl-Thomsen-nouveau (STn) and Macrophage-Galactose-Lectin (MGL) using GLYVAR ^®^ Ovarian I and II assays (Uniogen), conventional CA-125 (CanAg CA125 EIA, Fujirebio) and HE4 EIA (Fujirebio).

**Main outcome measure:**

Ovarian cancer.

**Results:**

The CA-125-STn was associated with subsequent ovarian cancer (p=0.002). In the primary analysis restricted to cases within 5 years, the area-under-the-curve for CA-125-STn was 0.68; with moving window analysis suggesting retained predictiveness up to 5-7 years. Of the cases diagnosed within 5 years, 27% were in the top 5% of CA-125-STn levels (≥2.3 U/ml), with 35% and 49% of cases diagnosed in the top 10% and 20% respectively. Our findings were replicated by independent measurements of a separate aliquot from a subset of cases and controls. Neither CA-125, HE4 nor CA-125 glycovariants detected by MGL importantly predicted subsequent ovarian cancer.

**Conclusions and Relevance:**

CA-125-STn can detect ovarian cancer several years before clinical diagnosis. Further research is warranted on larger cohorts with sequential samples to determine the shape of the relationship between CA-125-STn and risk, the value of longitudinal sampling of CA-125-STn, and whether other markers could be combined with CA-125-STn to more accurately predict ovarian cancer risk.

**Key Points:**

**Question**

Can glycovariants of CA-125 detect ovarian cancer years before clinical diagnosis?

**Findings**

In a case-control study of 323 Swedish women followed longitudinally, the Sialyl-Thomsen-nouveau (STn) glycovariant of CA-125, but not conventional CA-125 or other markers, predicted ovarian cancer. Close to half of ovarian cancer cases within 5 years of blood sample occurred in the patients with the top 20% of CA-125-STn glycovariant levels.

**Meaning**

CA-125-STn can detect ovarian cancer several years before clinical diagnosis.

## Full Text Availability

The license terms selected by the author(s) for this preprint version do not permit archiving in PMC. The full text is available from the preprint server.

